# Role of Cockayne Syndrome Group B Protein in Replication Stress: Implications for Cancer Therapy

**DOI:** 10.3390/ijms231810212

**Published:** 2022-09-06

**Authors:** John R. Walker, Xu-Dong Zhu

**Affiliations:** Department of Biology, McMaster University, Hamilton, ON L8S 4K1, Canada

**Keywords:** Cockayne syndrome group B (CSB) protein, replication stress, fork reversal, fork restart, fork protection, mitotic DNA synthesis (MiDAS), telomere replication stress

## Abstract

A variety of endogenous and exogenous insults are capable of impeding replication fork progression, leading to replication stress. Several SNF2 fork remodelers have been shown to play critical roles in resolving this replication stress, utilizing different pathways dependent upon the nature of the DNA lesion, location on the DNA, and the stage of the cell cycle, to complete DNA replication in a manner preserving genetic integrity. Under certain conditions, however, the attempted repair may lead to additional genetic instability. Cockayne syndrome group B (CSB) protein, a SNF2 chromatin remodeler best known for its role in transcription-coupled nucleotide excision repair, has recently been shown to catalyze fork reversal, a pathway that can provide stability of stalled forks and allow resumption of DNA synthesis without chromosome breakage. Prolonged stalling of replication forks may collapse to give rise to DNA double-strand breaks, which are preferentially repaired by homology-directed recombination. CSB plays a role in repairing collapsed forks by promoting break-induced replication in S phase and early mitosis. In this review, we discuss roles of CSB in regulating the sources of replication stress, replication stress response, as well as the implications of CSB for cancer therapy.

## 1. Introduction

Accurate replication of genomic DNA is essential to ensuring faithful transmission of genetic information during cell division. DNA replication is initiated at multiple origins and up to thousands of replication forks are established at a given time in S phase [1,2]. Timely coordination of these replication forks is critical to ensure that the entire genome is replicated only once per cell cycle. However, DNA replication is frequently challenged by both endogenous and exogenous sources of genotoxic stress. Any event that leads to slowing or stalling in progression of DNA replication forks is collectively termed as replication stress. Eukaryotic cells have evolved multiple pathways to restart stalled forks, including dormant origin firing, replication fork repriming, translesion synthesis, template switching, and fork reversal. In cases where stalled forks are collapsed by nucleases to generate DNA double-stranded breaks (DSBs), restart of collapsed forks is dependent upon homology-directed repair. Stalled replication forks can also be rescued by converging replication forks. We refer readers to excellent reviews of the causes of replication stress and the general pathways used to combat it [3,4,5,6].

Cockayne syndrome group B (CSB) protein, which contains an N-terminal region, a central ATPase domain, and a C-terminal region, is conserved from yeast to humans, with the greatest similarity found in the core ATPase domain. In humans, CSB is encoded by the human *ERCC6* gene, and was first described for its role in transcription-coupled nucleotide excision repair (TC-NER) [7]. Germline mutations in the *ERCC6* gene are responsible for the majority of clinical cases of Cockayne syndrome [8,9], which is characterized by severe photosensitivity, severe impairment of physical development, progressive neurological degeneration, cataracts, hearing loss, and segmental premature ageing. These phenotypes cannot be fully explained by a deficiency in TC-NER. Indeed, aside from TC-NER, CSB also participates in a variety of other cellular processes, including transcription [10,11,12], oxidative damage [13], aldehyde damage [14], DNA double strand break repair [15,16,17], telomere maintenance [18,19], mitochondria function [20], and cell division [21]. Recently, CSB has been reported to both possess an intrinsic fork reversal activity and play a role at stalled forks [22]. This review will focus on this novel role of CSB in the replication stress response. Readers are referred to recent excellent reviews on other functions of CSB [23,24,25,26].

## 2. CSB and Chromatin Remodeling Activity

CSB belongs to the SNF2 family, the defining feature of which is a bi-lobal ATPase domain composed of two Rec-A like domains that were originally identified in helicases [27]. Based on the similarities in the conserved helicase motifs, the proteins were divided into subfamilies. Within the SNF2 subfamily, several proteins including CSB function as ATP-dependent DNA translocases. While unable to separate nucleic acid strands as helicases do, they instead use ATP hydrolysis to move along DNA, in the process displacing proteins and effecting DNA conformation. This chromatin remodeling activity is highly regulated, as unrestrained remodeling activity could result in genotoxicity and loss of energy supply. Accordingly, SNF2 subfamily members contain other regions that are thought to provide regulatory functions. Defined domains such as bromodomain, HAND/SANT, chromodomain and macrodomain are present in the N- and C-terminal regions of some members, imparting functions such as nucleosome and protein partner binding, as well as contributing to activation of ATPase and translocase activity (reviewed in [4,28,29]). Some of these regulatory domains allow the chromatin remodelers to read the epigenetic code, while in other cases the remodelers themselves or their subunit partners contain domains that allow the rewriting of the histone code to recruit other proteins as necessary. Post-translational modifications likely contribute to the regulation of SNF2 subfamily remodelers, with CSB being one of the better-studied proteins in this regard (Table 1). 

**Figure 1:**
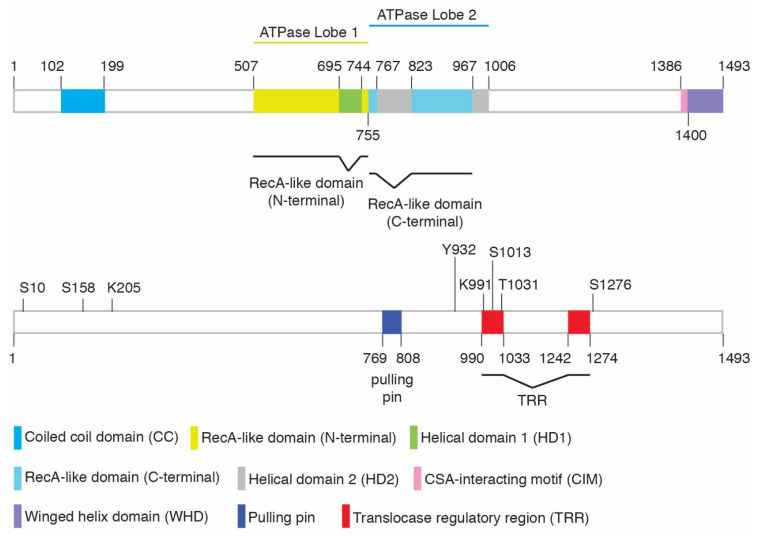
Schematic diagram of CSB.

Aside from the central ATPase domain, CSB contains a coiled-coil domain in its N-terminal region, a DNA translocase regulatory region (TRR), a CSA-interacting motif, and a winged-helix domain (WHD) at its C-terminus [32] (Figure 1). We refer readers to Cui et al. (2022) for a more detailed sequence and structural analysis [32]. While CSB has been reported to interact with a variety of proteins [39,40], it is not considered to be part of a constitutive complex, nor does it contain well-characterized protein binding domains other than its C-terminal winged-helix domain (WHD). The WHD binds ubiquitin [16,41,42] and mediates the interaction of CSB with BRCA1, MRE11/RAD50/NBS1, and RIF1 [16,33]. The best-known interaction is that of CSB with the stalled RNA polymerase II complex. In the cryo-EM structures of this complex [43], the CSB ATPase domain primarily makes contacts with DNA and RNA, but also makes contacts with the RNA polymerase II clamp and protrusion as well as with CSA. The CSA interaction motif (CIM) of CSB [44] is shown to interact with CSA, but other domains such as the coiled-coil domain and the WHD are not visible, likely due to flexible linkages between domains. Based on the Alphafold predicted structure (AF-Q03468), approximately 50% of CSB appears to be disordered and yet may play important roles.

The ATPase activity of CSB is stimulated by double-stranded DNA (dsDNA), dsDNA combined with single stranded DNA, as well as nucleosomal DNA [45,46]. CSB interacts with core histones through histone tails, rearranges nucleosomes in vitro [47], and evicts histones in vivo in a manner dependent upon its ATPase activity [16]. Regulation of the ATPase activity of CSB may involve post-translational modifications as well as protein–protein interactions. Work done in the Fan lab has revealed that CSB is capable of autoinhibition of its ATPase activity by its N-terminal region [48], while our work has demonstrated that this N-terminal inhibition could be alleviated in vivo through phosphorylation at S10 and S158 [16]. S10 sits at a long unstructured region at the N-terminus, while S158 is positioned between two helices that comprise the coiled-coil domain. CSB is also phosphorylated on several sites in the C-terminal region. Phosphorylation at T1031 and S1276 affects interaction of CSB with binding partners MRE11 and BRCA1, respectively [22,33], while phosphorylation of S1013 helps to regulate CSB’s translocase activity [32]. CSB has been reported to interact with the c-Abl tyrosine kinase and to be phosphorylated by this kinase at Y932 in response to oxidative damage [31]. The c-Abl kinase, which acts downstream of both DNA-PK and ATM kinases, helps to regulate the DNA damage response and to control cell fate [49]. It has been suggested that CSB regulates the auto-phosphorylation and activation of c-Abl in response to oxidative damage [31]. Previously, we have reported that knockout of CSB impairs ATM- and CHK2-mediated DNA damage response [15]. SUMOylation and ubiquitylation have also been shown to regulate CSB function [36,37,42,50] (Table 1). 

Ubiquitin binding by the WHD has been shown to be important for CSB function in TC-NER and oxidative damage repair [37,41,42]. Sensitivity to cisplatin, a chemotherapeutic drug that can cause replication stress, has also been shown to increase upon mutating amino acids in the WHD critical for ubiquitin binding, although other CSB functions such as promotion of RNAPII occupancy at promoter pause sites (PPP) appear to be dependent upon other regions in this domain [11]. Ubiquitylation plays critical roles in the regulation of DNA double strand break repair as well as in the replication stress response, yet specific proteins that are targeted by CSB via its WHD-mediated ubiquitin binding have yet to be elucidated. 

PARylation has also been shown to play critical roles in DNA repair and the replication stress response. Known to bind single stranded DNA breaks, PARP1, the most active PARylation enzyme, is thought to PARylate itself and other proteins upon binding to single stranded DNA breaks (SSBs), recruiting proteins, especially those involved in HR, required for subsequent repair (reviewed in [51]). In the replication stress response, PARP1 serves as an inhibitor to RECQ1 to ensure that repair has been completed before replication restart can begin [52]. PARP1 has been reported to act epistatically with CSB during repair of DNA base damage [53]. CSB has been shown to be retained at sites of DNA damage by binding to PAR-polymers, including auto-PARylated PARP1, and to subsequently remove PAR from DNA following DNA repair [54,55,56]. Two PAR binding sites have been characterized in the unstructured N-terminal region [55]. As PARP inhibitors have been approved for cancer therapy in a variety of cancers [57], understanding the interactions between PARP1 and CSB is of significant interest. Further studies examining the interaction between CSB and PARP1 in the replication stress response are warranted.

## 3. CSB-Deficient Cells and Their Sensitivity to Replication Stress-Inducing Agents

There are a variety of endogenous sources of replication stress, including abasic sites, DNA bases with modifications (e.g., oxidation, methylation, DNA crosslinks), DNA with secondary structures (e.g., G-quadruplexes, hairpin), DNA-RNA hybrids (R-loops), repetitive sequences with few replication origins, DNA-protein adducts, collision between DNA replication and transcription, and oncogene activation [4,6,58,59]. Replication stress can also be induced by external sources of genotoxic stress, including UV radiation, which induces DNA intrastrand crosslinks; ionizing radiation (IR), which induces both base damages and DNA breaks; as well as a variety of chemical compounds. These chemical compounds include, but are not limited to, topoisomerase inhibitors such as camptothecin (CPT) and etoposide (ETP), which induce DNA breaks; PARP inhibitors such as olaparib, rucaparib, niraparib and talazoparib, which are thought to trap PARP onto DNA; DNA crosslinking and methylating agents such as cisplatin, mitomycin C (MMC) and methyl methanesulfonate (MMS); inhibitors of ribonucleotide reductase such as hydroxyurea (HU); and inhibitors of DNA polymerases such as aphidicolin (APH) [60].

CSB deficiency was first described as rendering cells hypersensitive to UV [7], which induces the formation of thymidine dimers that block transcription as well as DNA replication. Since then, numerous studies have revealed that CSB deficiency also sensitizes cells to IR and a wide range of chemical compounds that induce DNA replication stress, including crosslinking agents, methylating and oxidizing agents, SSB- and DSB-inducing agents, and nucleotide analogs (Table 2), underscoring an important role of CSB in promoting cell survival in response to replication stress. 

## 4. Association of CSB with Replication Forks in Unperturbed Conditions

**Figure 2:**
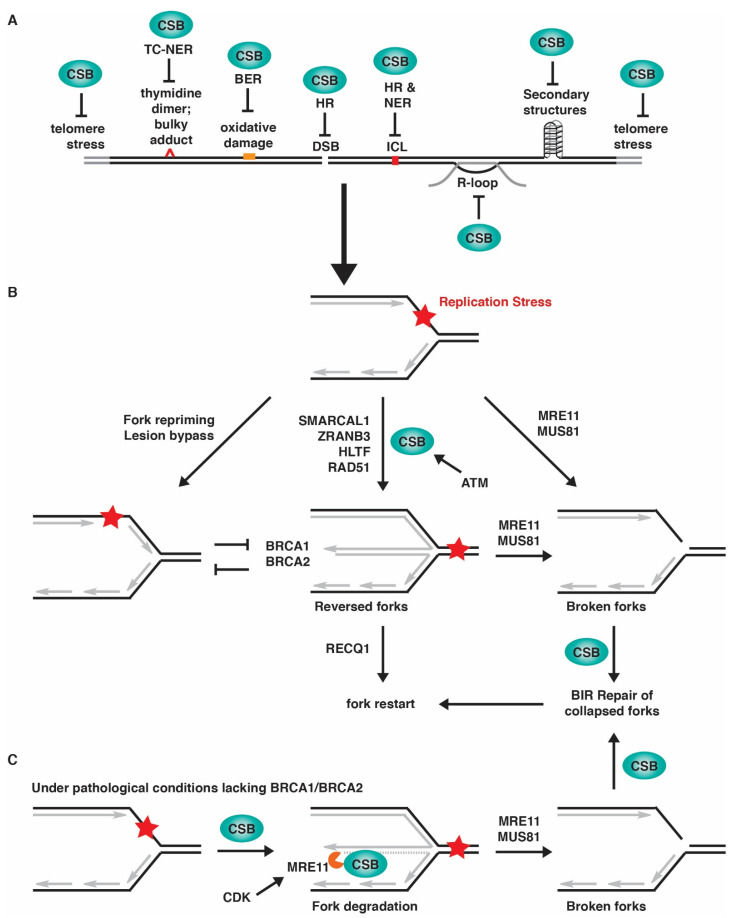
CSB regulates replication stress through multiple mechanisms. (**A**) CSB suppresses a variety of sources of replication stress. (**B**) CSB promotes fork reversal, and this activity is dependent upon its ATPase activity, which is controlled by ATM-dependent phosphorylation of CSB on S10 in vivo. CSB also promotes BIR-mediated repair of collapsed forks to facilitate fork restart. (**C**) Under pathological conditions lacking BRCA1 or BRCA2, CSB promotes fork degradation through both catalyzing fork reversal and recruiting MRE11 to stalled forks. The latter is controlled by CDK-dependent phosphorylation of CSB on T1031.

Independent proteomic analyses of proteins purified by iPOND (isolation of proteins on nascent DNA) and NCC (nascent chromatin capture) have revealed the association of CSB with newly replicated DNA [77,78]. In addition, using a proximity ligation assay (PLA)-based in situ protein interactions with nascent DNA, known as SIRF [79], CSB is found to interact with EdU-labeled nascent DNA [22], lending further support to the notion that CSB is associated with ongoing replication forks. It is likely that CSB facilitates DNA replication under normal conditions by resolving intrinsic obstacles through multiple mechanisms (Figure 2A). CSB could eliminate endogenous sources of DNA damage to support progression of DNA replication forks since CSB is engaged in a variety of DNA repair pathways. In addition, CSB could facilitate progression of DNA replication forks through regions of the human genome that are difficult to replicate. Loss of CSB has been reported to promote chromosome fragility associated with tandemly repeated genes for small RNA, common fragile sites (CFSs), and telomeres [18,19,32,80]. Moreover, CSB could support progression of DNA replication forks through non-canonical DNA secondary structures since CSB has been reported to participate in resolution of R-loops [81] and transcription-linked secondary DNA structures [82]. Lastly, CSB could support progression of DNA replication forks by reducing collisions between transcription and replication. Loss of CSB impairs RNA polymerase II (RNAPII) pausing at promoter proximal pause (PPP) sites [11], a key step in the regulation of transcription elongation at protein coding genes [83]. These findings underscore an important role of CSB in facilitating DNA replication under normal conditions. Indeed, primary cells derived from CS patients lacking functional CSB exhibit defects in telomere replication and enter premature senescence [19]. 

## 5. Association of CSB with Replication Forks in Response to Replication Stress

Analysis of differential salt extraction of chromatin has revealed that CSB is largely soluble in nucleoplasm under normal conditions but becomes associated with chromatin in cells exposed to replication stress-inducing agents such as UV, CPT, and ETP [15,48]. While CPT and ETP induce DNA double-stranded breaks, UV induces bulky lesions [60]. Interestingly, CPT- and ETP-induced chromatin association of CSB persists even 8 h post CPT and ETP treatment, whereas UV-induced chromatin association of CSB peaks 2 h post UV, and is largely lost 4 h post UV treatment [15]. This difference in the kinetics of CSB association with chromatin following treatment with CPT and ETP versus UV may reflect the differential roles of CSB in DNA double-stranded break repair [15,16,33] and transcription-coupled nucleotide excision repair [7]. The use of the differential salt extraction of chromatin method to examine chromatin association of CSB following exposure to replication stress is limited since it fails to detect HU-induced association of CSB with chromatin (J.R. Walker and X.-D. Zhu, unpublished data). A more sensitive PLA-based SIRF analysis reveals a significant increase in the interaction of CSB with EdU-labeled nascent DNA in cells exposed to HU [22], suggesting that CSB is recruited to HU-induced stalled forks. This recruitment relies on CDK-dependent phosphorylation of CSB on T1031, which also mediates the CSB-MRE11 interaction [22], suggesting that this interaction is necessary for CSB recruitment to stalled forks. 

## 6. CSB and Its Role in Fork Reversal in Response to Replication Stress

In mammalian cells, a commonly used mechanism in response to replication stress is fork reversal, which involves the coordinated annealing of newly-synthesized DNA strands, leading to the formation of four-way structures, also known as a “chicken foot” structure [84,85]. Fork reversal is thought to serve as a protective mechanism, allowing resumption of DNA synthesis without chromosome breakage [85,86]. Fork reversal may provide the stability for a stalled fork until it is rescued by a converging fork from a distal origin [6,85]. Fork reversal may also facilitate repair of DNA lesions by placing them in the context of double-stranded DNA. Furthermore, fork reversal may allow nascent DNA strands to be engaged in template switching, thereby bypassing lesions to restart DNA replication. However, fork reversal can become a source of replication stress when DNA damage is excessive, or fork protection is compromised under pathological conditions such as deficiency in BRCA1 and BRCA2 [87,88,89]. In this situation, reversed forks are degraded by MRE11 and EXO1 nucleases or cleaved by MUS81 [90,91,92].

Biochemical assays using recombinant proteins and synthetic forked substrates are powerful tools that can be used to directly examine the fork reversal activity in vitro. SMARCAL1, HLTF, FANCM, and RAD54 are found to catalyze fork reversal in vitro [93,94,95,96,97]. Using in vitro biochemical assays, our work has shown that CSB possesses an intrinsic ATP-dependent fork reversal activity albeit only when the protein lacks portions of the N-terminal domain [22]. CSB’s N-terminal region auto-inhibits its ATPase activity both in vivo and in vitro [16,48]. It is likely that CSB catalyzes fork reversal in vivo under the conditions where its N-terminal inhibition is released. Phospho-mimic mutations at S10 and S158 were unable to relieve the N-terminal inhibition in vitro, suggesting that in cells relief of this inhibition may require interactions with other factors. CSB-mediated fork reversal is also dependent upon its ATPase activity, as there was no significant increase in fork reversal observed for the ATPase-dead CSB mutant or for wild type (WT) CSB in the absence of ATP [22]. CSB has also been reported to promote fork annealing and strand exchange in vitro [98], an activity that could also play a role in fork reversal and repair.

Electron microscopy (EM) is a powerful technique that allows for direct visualization of DNA replication fork intermediates [99]. Using EM, fork reversal is found to occur in about 15–30% of replication forks in cells exposed to a variety of replication stress-inducing agents [100]. EM has been used to demonstrate the in vivo role of several chromatin remodelers in fork reversal, including SNF2 family members HLTF and ZRANB3, as well as the recombinase RAD51 and FBH1 [87,100,101,102,103]. Aside from EM, fork reversal in vivo can also be indirectly measured through analyses of fork progression and fork protection using single molecule-based DNA fiber assays. It has been reported that fork reversal slows down fork progression in response to mild replication stress [100,101,103], which can be prevented by depletion of known fork reversal factors such as SMARCAL1, ZRANB3 or HLTF [87,93,94,101]. In addition, it has been reported that fork reversal is a prerequisite for MRE11-dependent fork degradation in BRCA1/2-deficient cells [87,89,91].

Although the role of CSB in fork reversal has not been directly demonstrated by EM in vivo, loss of CSB is found to prevent slowdown in fork progression in cells exposed to a low level of replication stress of 50 μM hydroxyurea [22], similar to depletion of SMARCAL1, ZRANB3 or HLTF. In addition, CSB is found to promote MRE11-mediated fork degradation in BRCA1/2-deficient cells in a manner that is dependent upon both its ATPase activity and its phosphorylation on S10 [22]. CSB is phosphorylated by ATM on S10 and this phosphorylation helps release the autoinhibition of CSB’s N-terminal region on CSB’s ATPase activity [16]. These in vivo findings, combined with the in vitro finding that CSB possesses an intrinsic fork reversal activity, suggest that CSB is a novel SNF2 protein capable of catalyzing fork reversal and that CSB’s fork reversal activity is likely controlled by ATM in vivo (Figure 2B). 

Aside from BRCA1 and BRCA2, CtIP and BODL1 have also been implicated in fork protection, since they inhibit DNA2-mediated degradation of nascent DNA strands [104,105]. While depletion of SMARCAL1, ZRANB3 or HLTF abolishes DNA2-mediated fork degradation in CtIP-depleted cells [105], loss of CSB has no effect on fork degradation in either CtIP- or BOD1L-depleted cells [22]. These findings highlight an important distinction between CSB and other fork remodelers at stalled forks under pathological conditions. Interestingly, in cells with deficiencies in either BRCA1 or BRCA2, CSB recruits MRE11 to stalled forks and it has been suggested that CSB promotes fork degradation in BRCA1/2-deficient cells through both catalyzing fork reversal and recruiting MRE11 to stalled forks [22] (Figure 2C).

## 7. CSB and Restart of Stalled Forks

Mammalian cells have evolved multiple mechanisms to restart stalled replication forks, including fork reversal, replication re-priming downstream of a DNA lesion, lesion bypass through the use of either translesion synthesis (TLS) polymerases or template switching (TS), and dormant origin firing (see review in [5,6]). A stalled fork can also be rescued by a converging fork traveling from a neighbouring origin. CSB has been implicated in catalyzing fork reversal [22], which may allow time for the cell to repair the damaged DNA using a variety of mechanisms depending on the nature of the lesion. As a multifunctional DNA repair protein involved in base excision repair (BER), homologous recombination (HR), TC-NER, and interstrand crosslink (ICL) repair [23], CSB can be expected to assist in the repair of DNA lesions in the context of reversed forks. It has been suggested that the reversed forks may need to be remodeled back into the three-way junction to permit fork restart, a process that is dependent upon RECQ1 but inhibited by PARP1 [52]. Given the reported interplay between CSB and PARP1 [55], it would be of interest to examine whether CSB helps regulate RECQ1-mediated fork restart. Repriming of replication forks downstream of a DNA lesion is mediated by the DNA-directed primase/polymerase PRIMPOL [106,107]. Replication repriming competes with fork reversal and generates post replicative gaps that are subsequently filled in by either TLS or TS [6,86]. Suppression of fork reversal by depleting/deleting fork remodelers such as HLTF and SMARCAL1 promotes replication fork repriming, leading to accumulation of ssDNA gaps [103,108,109]. Similar to HLTF, SMARCAL, and ZRANB3, CSB deficiency leads to unrestrained fork progression in response to mild replication stress [22], which is dependent upon PRIMPOL and associated with accumulation of ssDNA gaps (N.L. Batenburg and X.-D. Zhu, unpublished data), further supporting the notion that CSB is a fork reversal protein. CSB is not thought to be involved in dormant origin firing [22], but whether CSB plays a role in lesion bypass through the use of either TS or TLS remains to be determined. 

Prolonged stalling of DNA replication can lead to fork incision on stalled or reversed forks, resulting in broken forks that need to be repaired before restarting DNA synthesis. Fork incision is generally carried out by structure specific endonucleases such as MUS81 and EEPD1 (reviewed in [110,111]). Incision of stalled forks leads to one-ended DNA double-stranded breaks (DSBs), which are thought to be repaired by homology-directed break-induced replication (BIR) [112]. CSB regulates both HR and BIR repair of DSBs [15,16,17,22,33]. Our recent work suggests that CSB facilitates BIR-mediated restart of stalled forks (Figure 2B) and that this function of CSB promotes survival of cancer cells with deficiencies in either BRCA1 or BRCA2 in response to replication stress [22]. MUS81 is thought to depend upon nicking by MRE11 before incising the DNA [90], and in accordance with this finding, CSB-mediated fork restart is dependent upon MRE11 [22] (Figure 2B). Chromatid breaks and radial chromosomes arise from replication stress in BRCA1/2-deficient cells in a manner dependent upon MRE11 and MUS81 [90]. Loss of CSB further exacerbates chromatid break formation in BRCA1/2-deficient cells in response to replication stress [22], suggesting that in the absence of CSB, fork incision occurs but repair of broken forks is compromised. Interestingly, loss of CSB mitigates the formation of radial chromosomes in BRCA1/2-deficient cells in response to replication stress, which is unlikely due to a defect in NHEJ [22]. How CSB mediates radial chromosome formation in BRCA1/2-deficient cells in response to replication stress remains to be determined. 

## 8. CSB and MiDAS

Certain regions of the human genome are intrinsically difficult to replicate, such as common fragile sites (CFSs). CFSs are prone to forming gaps and breaks on metaphase chromosomes upon exposure to a low dose of aphidicolin, the DNA polymerase inhibitor, in S phase [113]. They frequently remain under-replicated in S phase, and their replication continues throughout G2 and into early mitosis [114,115]. DNA synthesis at CFSs in mitosis relies on a DNA repair process known as mitotic DNA synthesis (MiDAS) (reviewed in [116,117]). MiDAS is thought to be a form of RAD52- and POLD3-dependent break-induced replication. It has been suggested that RAD52 acts at an early step in MiDAS by mediating DNA annealing from a collapsed replication fork into regions of microhomology, followed by POLD3-dependent DNA synthesis [118]. Stalled replication forks that persist into mitosis are thought to be cleaved by MUS81-EME1-SLX4 and RECQ5 has been implicated in removing RAD51 from stalled forks to facilitate CFSs cleavage by MUS81 [119]. A compromise in MiDAS leads to a marked increase in aberrant chromosome segregation events in mitosis, including ultrafine bridges (UFBs), chromatin bridges and lagging chromosomes as well as micronuclei and 53BP1 bodies in the subsequent G1 phase [120,121].

**Figure 3:**
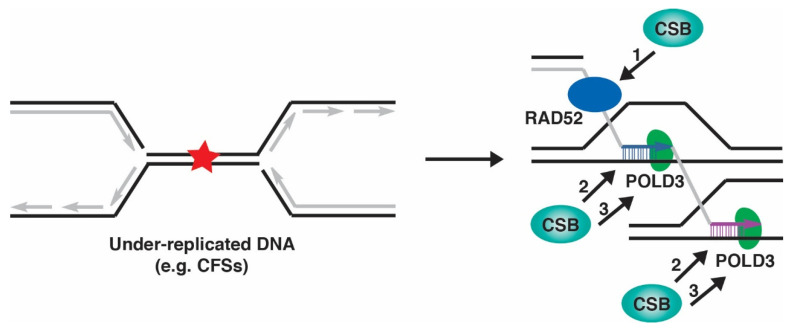
CSB promotes MiDAS through several possible mechanisms. CSB could act in parallel to facilitate RAD52-mediated strand annealing (arrow 1). CSB could prepare DNA substrates suitable for POLD3-mediated DNA synthesis (arrow 2). CSB could recruit POLD3 to sites of DNA synthesis to promote MiDAS (arrow 3).

Our recent work has demonstrated that CSB promotes MiDAS, as evidenced by a pronounced reduction in EdU incorporation at CFSs during prometaphase in CSB knockout (KO) cells compared to CSB wild type cells [32]. The role of CSB in this process is epistatic to that of RAD52, as treatment with a RAD52 inhibitor AICAR resulted in an equivalent reduction of EdU incorporation between CSB-WT and CSB-KO cells. While dispensable for recruitment of RAD52 and MUS81 to CFSs, CSB promotes aphidicolin-induced POLD3 foci in early mitosis [32]. POLD3 is thought to act downstream of MUS81 and RAD52 [118]. These findings taken together suggest that CSB acts in parallel or downstream of RAD52 and MUS81 to promote POLD3 in MiDAS. How CSB regulates POLD3 in MiDAS remains to be determined, but there are a couple of possible mechanisms to be considered (Figure 3). CSB could interact with POLD3 and recruit it to CFSs in MiDAS. Alternatively, CSB could prepare a substrate suitable for POLD3 in MiDAS, perhaps by assisting RAD52 in strand annealing or displacing nucleosomes. 

CSB is dependent upon its ATPase activity to promote MiDAS, as overexpression of the CSB ATPase-dead W851R mutant fails to rescue EdU incorporation in CSB-KO prometaphase cells following exposure to aphidicolin [32]. CSB is also dependent upon its DNA translocase activity to promote MiDAS since mutations in both the pulling pin (F796A) and the recently defined translocation regulatory region (TRR) (F1005A; HD1268AD1269A; S1013A) abrogate the ability of CSB to rescue EdU incorporation in CSB-KO prometaphase cells following exposure to aphidicolin (Figure 1) [32]. S1013 (S^1013^Q) is a potential ATM/ATR phosphorylation site, suggesting that the translocase activity of CSB in MiDAS is likely controlled by ATM/ATR. In agreement with the role of CSB in MiDAS, loss of CSB leads to a pronounced increase in the formation of UFBs, micronuclei, and 53BP1 nuclear bodies in G1 daughter cells following treatment with aphidicolin [32]. An accumulation of micronuclei, multinucleated cells, and chromatid bridges has previously been observed in CSB-deficient patient cells expressing the CSB K991R ubiquitylation site mutant (within TRR) in the absence of exogenous replication stress [37], attesting to the importance of CSB in the maintenance of replication fidelity.

## 9. CSB and Telomeres

Telomeres are specialized heterochromatic structures found at the ends of linear eukaryotic chromosomes. Human telomeric DNA consists of tandem repeats of TTAGGG, which are bound by a telomere-specific complex known as shelterin [122,123]. The function of shelterin proteins is highly regulated by post-translational modifications [124]. Loss of shelterin proteins can lead to disruption in telomere protection and telomere length maintenance [123]. Cancer cells must maintain their telomere length for their sustained proliferation, and they do so by either activating telomerase, a ribonucleoprotein complex, or by utilizing a HR-based recombination pathway, referred to as alternative lengthening of telomeres (ALT). Both telomerase and ALT are targets in cancer therapy [125]. CSB has been reported to promote both telomerase-dependent and ALT-mediated telomere maintenance [18,19], making CSB an attractive anti-cancer target. 

CSB plays an important role in resolving replication stress at telomeres in both telomerase-expressing and ALT cells as evidenced by increased levels of telomere fragility (also known as telomere doublets) observed in the absence of CSB [18,19]. CSB regulates the homeostatic level of TERRA, non-coding telomere repeat containing RNA, in both telomerase-positive and ALT-positive cells [19], suggesting that CSB could limit the formation of transcription-associated telomeric R-loops. Telomeric DNA is prone to adopt G4-DNA structures [126]. CSB could also resolve telomere G4-DNA formation since it has been implicated in resolution of non-B DNA including G4 DNA both in vitro and in vivo [82,127]. Furthermore, CSB could support progression of replication forks through telomeres by utilizing its demonstrated activities in catalyzing fork reversal and promoting restart of stalled forks [22,32].

Replication stress at telomeres is elevated in ALT cancer cells compared to telomerase-expressing cancer cells [125]. Recent work from our lab has demonstrated that CSB promotes telomeric recruitment of a number of proteins engaged in homology-directed repair, including MRE11, NBS1, BRCA1, BLM, RPA, and POLD3 in ALT cells [18]. While BRCA1 is key to HR [128], POLD3 is a core protein of BIR [129]. CSB has been implicated in both HR- and BIR-mediated repair of DSBs [15,16,17,22,33]. These findings suggest that CSB promotes both HR- and BIR-mediated repair pathways to suppress telomere replication stress in ALT cells. Depletion of SMARCAL1, which has been implicated in suppressing replication stress at ALT telomeres [130], further exacerbates telomere fragility in CSB-KO ALT cells [18], suggesting that the activity of CSB at ALT telomeres is not epistatic but rather synthetic to that of SMARCAL1. This synergistic relationship between CSB and SMARCAL1 is likely to be extended beyond telomeres since depletion of SMARCAL1 also exacerbates the formation of chromatid breaks in CSB-KO cells [18]. Depletion of SMARCAL1 and loss of CSB also act synergistically to affect cell proliferation [18].

## 10. CSB and Its Implications in Targeted Therapy in Cancer

Somatic mutations and copy number variations (CNVs) of CSB are present in a variety of cancers according to cancer genomic databases of both cBioPortal and the Catalog of Somatic Mutations in Cancer (COSMIC). Cancer-associated CSB mutations have been reported to impair the ability of CSB to promote chemoresistance [11]. CSB has been implicated in cancer development, cancer progression, and cancer treatment (reviewed in [26,131]). CSB is a multifunctional protein that can be used by cancer cells to contribute to continued growth. CSB is required for maintaining telomere stability in ALT cells [18], which comprise approximately 10–15% of human cancers [132]. CSB plays an anti-apoptotic role by regulating p53 in response to DNA damage or hypoxia [133]. This function is in addition to direct roles of CSB in DNA repair through such mechanisms as TC-NER, BER, HR, and ICL repair, which could alleviate the impact of cancer therapeutic drugs. Cancer cells, including those from colorectal, breast, ovarian, lung, kidney and extra-cranial solid tumours, have been reported to overexpress CSB [65,69,131,134,135]. One mechanism by which lung cancer cells can overexpress CSB is through the downregulation of the miRNA tumour suppressors let-7 and miR-29 [69]. An integrative genomic analysis demonstrated that CSB expression is upregulated in breast cancer, and that there is a 30–300% increased risk of breast cancer conferred by six different ERCC6 variations, one of which is in the 3′ UTR and may affect miRNA binding [135]. Increased expression of CSB correlates with poor survival in colorectal patients and chemoresistance [134]. Furthermore, our recent analysis of BRCA1 mutation-bearing breast cancer patients demonstrates that increased expression of CSB is associated with reduced survival as well as reduced relapse free time compared to those with reduced CSB expression [22]. These findings taken together suggest that CSB plays key roles necessary for cancer cell survival and that inhibiting CSB could be beneficial in targeted therapy. Since many chemotherapeutic drugs are directed at inducing replication stress, our recent finding that CSB directly plays a role in this process further raises its profile as a chemotherapeutic target.

Many approved chemotherapeutic drugs such as cisplatin, camptothecin (CPT), olaparib, 5-AZA-2′-deoxycytidine (5-azadC), oxaliplatin, carboplatin, and 5-fluorouaracil (5-FU) have been shown to cause sensitivity in CSB-deficient cells (Table 2). The loss of CSB function in TC-NER, ICL-repair, and BER throughout the cell cycle is likely to increase the stress during replication arising from these compounds where cytotoxicity is thought to occur. The toxicity of CPT is replication dependent, since aphidicolin-induced inhibition of DNA synthesis abolishes its toxicity [62]. Similarly, treatment with aphidicolin reduces chromosome damage following exposure to 5-azadC, an inhibitor of DNA methyltransferase 1 (DNMT1), suggesting that trapping of DNMT1 at replication forks leads to replication fork collapse [136]. Increased levels of DNA damage have been observed in CSB-deficient cells exposed to 5-azadC, which is thought to be due to a failure in CSB-mediated TC-NER, leading to increased replication stress [64]. The synergistic sensitivity observed in a panel of acute myeloid leukemia (AML) cells exposed to olaparib and 5-azadC has been ascribed to a failure to repair the 5-azadC-induced lesions through BER [137], which could also be a mechanism behind the sensitivity of CSB-deficient cells to this drug since CSB also plays a role in BER. Taken together, these results suggest that loss of CSB function in repairing DNA lesions outside of replication further potentiates the damage chemotherapeutics cause during replication. Deficiencies in transcription- and replication-dependent pathways have been shown to be additive regarding cellular sensitivity to cisplatin [68]. It is likely that that some of the functions ascribed to CSB outside of S phase are also active during S phase. For example, CSB has been proposed to carry out ICL repair in a pathway dependent upon the structure-specific endonuclease SNM1A, during the G1 phase in replicating cells or in non-cycling cells [70]. SNM1A also participates in replication-dependent crosslink resolution, so it remains a possibility that CSB assists in this process [138]. Our finding that CSB contributes to BIR when BRCA1/2-deficient cells are exposed to replication stress-inducing chemotherapeutics shows that these cells depend upon CSB for survival [22]. Similarly, the CSB function in ALT maintenance allows these cancer cells to proliferate [18]. 

There are two main ways in which CSB could be used in chemotherapy: the first would be to use CSB as a biomarker, and the second would be to use an inhibitor of CSB to enhance tumour cell death in response to chemotherapy or endogenous sources of replication stress. In the first approach of using CSB as a biomarker, an increased expression of CSB would predict a reduction in the sensitivity of cancer cells to many chemotherapeutic compounds. If other known deficiencies, such as the loss of BRCA1/2, were also identified, an increased expression of CSB would predict an enhanced dependence of these cancer cells to BIR. Chemotherapies targeting this specific pathway would then likely be more efficient. An example would be inhibition of RAD52, for which a leading compound has been identified [139]. On the other hand, reduced expression of CSB would predict a favorable response of tumor cells to chemotherapy. In the case of tumor cells with deficiency in BRCA1 or BRCA2, loss of CSB expression would also predict that these cancer cells would not be able to depend upon BIR for survival and might turn to different salvage repair pathways such as alternative end joining, which in turn could be targeted. It has been proposed that in the absence of fork reversal, PRIMPOL-mediated repriming leads to ssDNA gaps that need to be filled, and chemosensitivity is enhanced in BRCA1/2-deficient cells when both fork reversal and gap filling are targeted [107]. Expression levels of CSB in these cells could then play a role in deciding whether to use this strategy. A greater understanding of the roles that CSB plays in various salvage repair pathways could be of great use when using CSB expression as a biomarker. Additionally, as CSB regulates telomere maintenance in both telomerase-positive and ALT cancer cells, a choice to target these pathways may be influenced by the levels of CSB expression.

The second approach involving targeting CSB to promote synthetic lethality in response to chemotherapeutic drugs requires the development of an inhibitor to CSB. The evaluation of CSB as a potential chemotherapeutic target has relied primarily on the use of anti-sense technology [65], which is likely to eliminate all functions of CSB. Small molecule-based inhibitors, on the other hand, could be developed to target specific functions of CSB, although targeting the ATPase activity might be the most straightforward. Indeed, a chemotherapeutic drug that could trap CSB on DNA without eliminating it, in much the same way that PARP inhibitors do, could be very helpful. Thus, it is important to recognize that the effect of a small molecule inhibitor of CSB is likely to be different from that of elimination of CSB. 

The paucity of inhibitors discovered to date amongst the broader SF2 helicase family suggests that development of a CSB inhibitor may not be straightforward [140]. The most obvious target would be its ATPase activity, yet due to the conserved nature of the ATP binding pocket among the SF2 family, the danger of cross-reactivity would be high. A high-throughput approach examining the ATPase activity of a truncated SNF2 remodeler BRM (also known as SWI/SNF-related, matrix-associated, actin-dependent regulator of chromatin subfamily A member 2, SMARCA4) was carried out using an ADP-Glo assay (a luminescent ADP detection system that can be used with batch plate processing to rapidly identify compounds interfering with the ATPase reaction), with promising hits followed by a differential scanning-fluorimetry (DSF) assay [141]. This two-step approach is required to weed out inhibitors that bind to the nucleic acid substrate or otherwise interfere with the primary assay [140]. Following inhibitor refinement, structural studies revealed that several of the top compounds bind an allosteric pocket near the ATP binding site, reducing the likelihood of cross-reactivity by binding to a less conserved site [141]. An advantage to this high-throughput approach examining ATPase activity is that novel sites outside of the ATP binding pocket could be discovered, while at the same time screening for compounds that target the ATP binding site.

Another promising approach is that utilized by the Kapoor laboratory to design a chemical inhibitor to the AAA protein spastin, in which the design of heterocyclic compounds was improved by testing iterations against wild-type and constructs with engineered mutations in the ATP binding site [142]. A limited set of kinase inhibitors could be used in an initial ATPase screen, instead of the random hit approach [140]. Information obtained from the mutational and structural analysis could then lead to the design of a more specific inhibitor. The resistance analysis during design (RADD) approach [143] could be used to design CSB inhibitors from the start to avoid acquiring drug resistance. Structures containing the ATPase domain of CSB have been determined as well as predicted, forming the basis for computational docking that will assist in hit improvement. Once in vitro analyses have been carried out demonstrating blockage of ATPase activity, as well as specificity, in vivo cell-based studies could be carried out demonstrating increased sensitivity to tumour cells to the inhibitor either by itself or in combination with other anti-cancer compounds. 

Examination of the effects of CSB inhibition would need to go beyond effects observed in cell culture, with mouse models as a potential next step. Interestingly, recently a spinal cord injury (SCI) mouse model looking at the effects of siRNA-mediated CSB inhibition revealed that loss of CSB led to reduced oxidative stress, inflammation, apoptosis, and cell senescence [144], suggesting that utility of a CSB inhibitor would likely extend beyond chemotherapy. However, it is well known that mouse models don’t recapitulate Cockayne syndrome (CS) [145]. The identification of CSB as a factor required to prevent endogenous formaldehyde toxicity and the development of a mouse model that more closely mimics Cockayne syndrome than previously obtained will likely be very useful in this regard [14]. Using this mouse model, which involves the knockout of aldehyde dehydrogenase 5 (ADH5) to unmask the greater dependence in humans upon CSB function in repair of formaldehyde DNA crosslinking, the authors of this study demonstrate that CSB down regulates the induction of the anorexic hormone GDF15 in proximal tubule cells. Anorexic weight loss is often observed during platinum-based chemotherapy, and inhibition of CSB, unless it could be directed specifically to tumour cells (a current topic in the chemotherapy field) [146], would likely lead to increased weight loss and poor patient outcomes. Fortunately, strategies are being developed to combat GDF15-mediated anorexia [147], but these results suggest that specific mouse models may be required to evaluate the utility of CSB inhibition as a cancer therapeutic.

## 11. Concluding Remarks

Replication stress is a known driver of genomic instability, an underlying hallmark of cancer [148]. Elevated replication stress is frequently observed during the development of many human cancers [149]. A common mechanism shared by many clinically approved chemotherapeutic drugs is to induce excessive replication stress in cancer cells, thereby killing them. CSB, a chromatin remodeler, participates in various DNA repair pathways that are key to mitigate sources of replication stress in cancer cells. CSB also regulates responses of cancer cells to replication stress, making it both a biomarker and a target in cancer treatment. Identification of genetic pathways, deficiencies of which are synthetic to loss of CSB will be crucial in targeted cancer therapy involving CSB inhibitors. 

## Figures and Tables

**Table 1:** Summary of post-translational modifications of CSB.

Modification	Amino Acids	ID of Site			Modifying Enzymes	Function	Cellular Phenotype *	References
		MS	MA	Ab				
Phosphorylation	S10	–	Yes	Yes	ATM	Relief of CSB’s N-terminal inhibition; regulation of CSB’s chromatin remodeling	Sensitivity to olaparib but not UV	[16,30]
	S158	Yes	Yes	Yes	CDK	Relief of CSB’s N-terminal inhibition; regulation of CSB’s chromatin remodeling	Sensitivity to olaparib but not UV	[16,30]
	Y932	Yes	–	Yes	c-Abl	Regulation of CSB localization following oxidative damage	–	[31]
	S1013	–	Yes	–	Possible ATM/ATR	Regulation of CSB’s DNA translocase activity; regulation of fork restart and MiDAS	Sensitivity to olaparib ** but not UV	[32]
	T1031	–	Yes	Yes	CDK	Regulation of the CSB-MRE11 interaction; CSB recruitment to stalled forks; MRE11 recruitment to stalled forks in BRCA1/2-deficient cells; fork degradation in BRCA1/2-deficient cells	No sensitivity to olaparib	[22]
	S1276	Yes	Yes	Yes	CDK	Regulation of MRE11/RAD50/NBS1- and CtIP-mediated end resection; recruitment of BRCA1-C to DSBs; cell survival in response to olaparib and CPT	Sensitivity to olaparib and CPT	[33,34,35]
SUMOylation	K205	–	Yes	Yes	SUMO-2/3	TC-NER and UV resistance	Sensitivity to UV	[36]
Ubiquitylation	K991	Yes	Yes	–		Oxidative damage repair; transcription regulation	Sensitivity to paraquat or potassium bromate but not UV or cisplatin	[37]
PARylation	–	–	–	–	PARP1	Mitochondria DNA damage sensing	–	[38]

ID: Identification method; MS: mass spectrometry analysis; MA: mutational analysis; Ab: antibody; “–”: Not available. *: The effect of loss of post-translational modification of CSB on cellular sensitivity to DNA damage-inducing agents; **: N.L. Batenburg and X.-D. Zhu, unpublished data.

**Table 2:** List of a variety of DNA damage-inducing agents to which CSB-deficient cells are sensitive.

Agents	Mechanisms	Approved Chemotherapy *	References
*Radiation*			
UV radiation	Intrastrand crosslink		[7]
Ionizing radiation	Oxidative damage, single-stranded breaks, double-stranded breaks	Broadly used	[15,54,61]
***Enzyme inhibitors***			
Camptothecin (CPT) (Analogs include topotecan and irinotecan)	Topoisomerase I inhibitor	Analogs used against ovarian cancer, cervical cancer, colorectal cancer, small cell lung cancer; pancreatic cancer	[15,33,62]
Etoposide (ETP)	Topoisomerase II inhibitor	Small cell lung cancer, testicular cancer	[15]
Olaparib	PARP inhibitor	Breast cancer, ovarian epithelial, fallopian tube or primary peritoneal cancer, pancreatic cancer, prostate cancer	[15,22,33]
3-aminobenzene (3-AB)	PARP inhibitor		[63]
3,4-dihydro-5[4(1-piperidinyl)butoxy]- 1(2H)-isoquinolinone (DPQ)	PARP inhibitor		[54]
5-AZA-2′-deoxycytidine (5-azadC) (also known as Decitabine)	Traps DNA methyltransferases (DNMTs)	Myelodysplastic syndromes (MDS) including chronic myelomonocytic leukemia	[64]
5-fluorouracil (5-FU)	Thymidylate synthase inhibitor	Breast cancer, colorectal cancer, gastric cancer, pancreatic cancer	[65]
***Crosslinking chemicals***			
Cisplatin	Intrastrand and interstrand crosslink	Bladder cancer, ovarian cancer, testicular cancer	[22,37,66,67,68,69]
Mitomycin C	Interstrand crosslink	Gastric and pancreatic adenocarcinoma, urothelial cancer	[65,68]
Formaldehyde	Interstrand crosslink		[14]
Trioxsalen	Interstrand crosslink		[70]
Carboplatin	Intrastrand and interstrand crosslink	Ovarian cancer	[69]
Oxaliplatin	Intrastrand and interstrand crosslink	Colorectal cancer, Stage III colon cancer	[65]
***Methylating chemicals***			
Methyl methanesulfonate (MMS)	DNA methylation		[71]
***Oxidizing chemicals***			
Paraquot	Oxidative damage		[37,61]
Hydrogen peroxide	Oxidative damage		[61]
Potassium Bromate	Oxidative damage		[37]
Methanedione	Oxidative damage		[56]
***Nucleotide analog***			
5-hydroxymethyl-2′-deoxyuridine (HmdU)	Thymidine analog		[71]
***Other chemicals***			
N-acetoxy-2- acetylaminofluorene (NA-AAF)	DNA adducts		[72,73,74]
Angelicin	Monoadducts		[70]
4-nitroquinoline-1-oxide (4-NQO)	Single-stranded breaks and bulky adducts		[73,75,76]

* Identification of cancer types approved for specific chemotherapies obtained from the National Cancer Institute (www.cancer.gov (accessed on June 30, 2022)).

## Data Availability

Not applicable.
